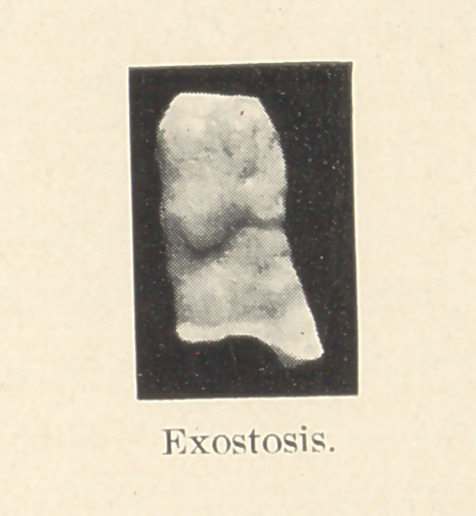# The Making of a Porcelain Filling

**Published:** 1903-06

**Authors:** N. S. Jenkins

**Affiliations:** Dresden, Germany


### THE MAKING OF A PORCELAIN FILLING.

BY N. S. JENKINS, D.D.S., DRESDEN, GERMANY.

Every step in making a porcelain filling should be taken with scrupulous exactitude.

### PREPARING THE CAVITY.

It is indispensable that the cavity should be shaped so that the matrix can be perfectly made and withdrawn without changing its shape in ever so slight a degree. To this we need not hesitate to sacrifice frail walls, especially upon occlusive surfaces, as the crowns of molars and bicuspids and the palatine walls of superior incisors. In all approximal cavities sufficient space must be gained by wedging or filing. At all occlusal surfaces the walls of the cavity must be at right angles, and thin edges should be avoided so far as possible. All the walls of the cavity should be as nearly at right angles as circumstances permit. Occasionally some decayed dentine can be allowed to remain until the completed filling is ready to be set, if

Exostosis.

its premature removal would necessitate undercuts; but usually every particle of decay can be removed with advantage in the first preparation of the cavity. All the edges should be clearly defined and well polished, that it may be possible to burnish the edges without injury to the matrix. It is well to polish also the interior of the cavity, as a smooth surface facilitates the removal of the matrix.

What instruments should be used to accomplish this perfect preparation of the cavity must depend upon the operator and the case. It is not sufficient for thorough treatment to simply have the cavity well shaped and polished. Every visible portion of the tooth under treatment must be examined, every bit of tartar must be removed, every pit and blemish in the enamel must be made as clean and smooth as practicable, and often the very shape of the tooth must be reduced to an ideal form. Chisels, files, sand-paper disks, drills of all shapes and sizes, corrundum wheels, disks, and points, polishing tape, diamond points, Scotch and Arkansas stones, spoon-shaped and other excavators, polishing powder, all serve their purpose according to the case and the feeling of the operator. It is only necessary to insist that, by whatever means may be found practicable, the cavity must be so prepared that it has that form which will make the filling secure; so cleansed that decay and discoloration cannot progress under the filling; so shaped that the matrix can be withdrawn without change; so perfect in edge that an exact joint can be obtained everywhere. This is possible in every cavity, but is sometimes not achieved except by the operator who takes his vocation seriously, and who thinks out every detail of every case as being to him, at that time, the most important thing in the world.

All this preparation is generally best done under the coffer-dam, and in some cases, where there is still room enough, it is well to take the matrix with the rubber dam in position. Ordinarily, however, it is preferable to take the matrix after having removed the dam. In such cases, if there is any bleeding of the gum, it may be stanched by the application of trichloracetic acid, a slight touch of the ten per cent. solution being usually sufficient. If blood or serum work underneath, the removal of the matrix is rendered more difficult, but the application of a thin film of vaseline to the edge of the gum and to the cavity seems to be unobjectionable; indeed, it facilitates the fixing of the matrix as well as its removal in all those cases where the cavity cannot be kept absolutely dry. If complete dryness is possible, the use of vaseline is superfluous; but in that large class of cases where the matrix must be carried over the gum, vaseline is indicated. Bruck uses olive oil instead of vaseline. Any oleaginous substance will probably answer the purpose if intelligently applied.

### TAKING THE MATRIX.

There are only two materials available, platinum-foil and gold-foil. Of the former it may be claimed that it is fairly adaptable and resists so high a degree of heat that tolerably refractory substances may be melted in it, even when it has not been embedded. The objections are, that no degree of skill can carry platinum-foil unbroken into very many cavities in obscure positions, that it cannot be burnished exactly over frail edges in numerous cases, nor always be made to conform to the general shape of the tooth where such conformity is desirable, and that its stiffness is so decided as to cause it sometimes to spring just enough to make a perfect joint impossible. None of these objections apply to gold-foil. With a little experience, the skill of any ordinary dentist is sufficient to carry gold-foil unbroken to the bottom of any properly shaped cavity, however obscure, and to burnish exactly over the edges of the cavity, with sufficient margin to give the shape of the tooth when such shape is desired; and the gold may be removed, by the exercise of a little dexterity, without the slightest change, as it possesses a docility which leads it to remain in that form into which it has been pressed. If then embedded in a soft asbestos paste, it remains unchanged during any number of heatings below its melting-point.

To the practised hand gold-foil No. 30 is sufficiently thick for all cavities. It should generally be annealed before using, the exceptions being in very small cavities or in very deep ones of moderate size. In these cases the objection to annealing is, that it renders the foil so cohesive that folds become somewhat intractable, and are not so easily reduced by burnishing. In large cavities this condition is less objectionable, and the matrix is made stiffer when worked after annealing, so that its removal without bending is more certain. The gold-foil should be cut of an appropriate shape and large enough to lap well over the edges in all directions, and should be carried first to the deepest part of the cavity with a small piece of amadou or cotton. For this purpose the ball-pointed tweezers, suggested by Hastings & Keyes, are very useful. After the foil has been pressed by successive pieces of amadou into every part of the cavity, it is well to remove this packing and follow it with disks of chamois skin, after the manner of Elander. Now also the edges should be burnished until everywhere a perfectly smooth margin has been secured. In forming the margins the rubber-capped instruments designed by Bruck are often very efficient, especially in crown and labial or buccal cavities. The packing can then be removed and the matrix gently coaxed out. If the cavity has been well shaped and the matrix properly made, this can always be done, with a little patience, by careful use of a hoe-shaped excavator. It is never necessary to fill the matrix with wax in order to remove it intact.

The gold matrix should always be embedded in a paste of powdered asbestos and water (even a platinum matrix is better managed if embedded), then placed in a platinum or a nickel-platinum cup, which should be exposed to a gentle heat until the paste has been completely dried. It is unnecessary, and usually objectionable, to add plaster or sand to the paste. Asbestos, of which there are many varieties, is a mixed silicate of magnesium and calcium. Care should be taken to select asbestos which, when exposed to the heat of the furnace, shows a sufficient proportion of silica to hold it together in a compact mass with a smooth surface.

### SELECTING THE COLOR.

There are two general principles to be considered. In approximal cavities it is better to select a shade slightly lighter than the natural tooth, and in labial and buccal cavities slightly darker. In all other cases it is well to conform to the natural color of the tooth so far as possible. Under all circumstances the color should be selected when the tooth is moist, and not when it has been bleached by dryness under the coffer-dam.

### MAKING THE INLAY.

It being granted that the gold matrix is preferable to one of platinum, for the reasons above mentioned, then a body fusing below the melting-point of gold must be selected for the inlay. Among such bodies, that known as the Jenkins Porcelain Enamel, being at the present time the one most commonly approved and adopted, on account of its great strength and density and the certainty with which it can be melted to an exact line and contoured to desire, is here selected to illustrate the process of making a porcelain inlay. The principles which control the manipulation of this material would, in general, apply to the use of any low-fusing body.

The matrix should be filled with the porcelain enamel powder, which has previously been well moistened with absolute alcohol. The mixing should be done with a perfectly clean spatula on an agate plate, since the powder is so hard that it will scratch and roughen either glass or porcelain plates, taking up undesirable particles and leaving, in time, a surface which cannot be kept clean. Alcohol is to be preferred to water because of cleanliness and ease of manipulation. It evaporates quickly and leaves a fairly solid mass in the matrix. It is well, from the beginning, to pack the powder to conform to the general shape desired in the finished filling.

The fusing is most accurately accomplished with the gas furnace designed for the material under consideration. The cup containing the matrix is held about midway in the furnace and a small flame from the gas-jet turned upon it at the same time with an air-blast from the foot-bellows. Sufficient air to make a blue flame should always be used, that there may be no unconsumed particles of carbon to discolor the furnace and possibly the filling. Slowly the heat should be increased until that point has been reached at which previous experiment has shown that the powder will just barely melt, and at this point it should be held until the desired flow is obtained. This often requires much patience. If the mass of the powder is unusually great, or an excess of asbestos has been used for embedding, one often tries to accelerate the melting process by employing too high a degree of heat. If that is done, the inlay will have an unnecessary degree of porosity. There is no material which cannot be made to boil and bubble by sudden and excessive heat, and when porcelain has been thus treated it is impossible to remelt it into a solid mass.

The great value of the process above described is that every stage can be seen, and the powder be melted each time exactly as is desired, until a practically homogeneous and solid mass is obtained. When the first fusing has been completed the cup can be at once removed from the furnace, the cover taken off, and the bottom of the cup wet with water to facilitate cooling. When cool, the matrix must be again packed and fused. Four such packings and meltings are necessary to obtain a perfect result. The first three meltings should stop just short of the most complete fusing, but the fourth, which is designed to exactly finish the edges and the contour, should be more thorough, being continued until the desired surface is obtained. In some complicated cases an even greater number of meltings are required, but generally four are sufficient.

When the inlay is completed it is well to let it cool slowly. When quite cool, the gold-foil, which may be wet with water to advantage, is to be removed by gently bending it back from the edges. When carefully done it may be removed in one piece. If any shreds of gold-foil remain sticking to the inlay, they may be removed with an excavator or a bur drill in the dental engine, or by boiling in *aqua regia.* The filling is now placed in the cavity and the fit proved. If hitherto each step has been taken aright, it will fit so exactly that no space can be seen, even with a powerful magnifying glass, between the filling and the cavity. Should there be slight overhanging edges, they may be removed with a sand-paper disk. This requires care and delicacy of touch, so as to remove only that which is superfluous.

The inner surface of the inlay will be found glazed, an undesirable condition for the attachment of cement. Elander and Reeves recommend removing this glaze with hydrofluoric acid. Great care should be exercised in using this powerful agent, which dissolves all metals except lead, gold, and platinum, and attacks all silicates. A drop placed upon the under surface of a porcelain inlay will speedily cause a roughness, intensified if the porcelain has not been solidly melted. The acid must in no case run over the edge. In those rare cases where a desirable depth of the cavity cannot be obtained, and where undercuts cannot therefore be easily made, this method is especially indicated. In most instances, however, the glaze can be removed by the use of a small sand-paper disk, and then it is well to make a few slight undercuts with a small diamond disk. These undercuts can be placed anywhere and add greatly to the security of the filling. Of course, they must be made with discretion, and not be used to weaken the inlay, nor should they be made without having inlay and disk kept cool with water, lest fracture ensue.

### SETTING THE INLAY.

The cavity should now, if practicable, be put under the coffer-dam, thoroughly dried, and a slight portion of the dentine nearly everywhere removed, so as to leave a very trifling space for the cement. In those cases where the dam cannot be applied, the gum may be touched with trichloracetic acid and the salivary ducts covered with napkins or absorbent cotton rolls, and the cavity can then be kept dry long enough to set the filling securely. A little oxyphosphate cement should be smeared upon the under surface of the inlay, as well as upon the floor of the cavity, and the filling then be gently but firmly pressed home. The cement must have a consistency appropriate to the case. If the case be a tooth with frail edges and a thin and delicate inlay, or one that is so complicated in shape that unusual care and deliberation must be exercised to put it into place, then the cement must be thinner than for a large cavity with strong edges. Under all circumstances every particle of superfluous cement must be pressed out. This may be done with tape, silk ligatures, wooden points, or any other means which may be indicated. It is important to have every cavity so shaped that there may be no question of where the filling should go and no doubt as to whether it is exactly in place. It should be held firmly in position until the cement has begun to crystalize, and then, when the pressure is relaxed, it is desirable to keep the tooth dry until the cement has well hardened. Dryness makes the tooth appear lighter in shade and, combined with the opacity of the cement, causes a momentary disappointment in color; but if the color has matched the moist tooth before setting, it will in a short time prove satisfactory after setting, when the tooth has had time to resume its normal color.

No other filling, except tin and gold, so increases in stability with advancing time as does porcelain. With each year the cement seems to gain in strength, until at last the filling can be removed only with extreme difficulty.

## Figures and Tables

**Figure f1:**